# Embracing the future of circular bio-enabled economy: unveiling the prospects of microbial fuel cells in achieving true sustainable energy

**DOI:** 10.1007/s11356-023-28717-0

**Published:** 2023-07-22

**Authors:** John Onolame Unuofin, Samuel Ayodele Iwarere, Michael Olawale Daramola

**Affiliations:** Department of Chemical Engineering, Faculty of Engineering, Built Environment and Information Technology, University of Pretoria, Private Bag X20 Hatfield, Pretoria, 0028 South Africa

**Keywords:** Sustainable energy, Circular bio-enabled economy, Microbial fuel cell

## Abstract

Sustainable development and energy security, highlighted by the United Nations Sustainable Development Goals (SDGs), necessitate the use of renewable and sustainable energy sources. However, upon careful evaluation of literature, we have discovered that many existing and emerging renewable energy systems (RESs) prioritize renewability over true sustainability. These systems not only suffer from performance inconsistencies and lack of scalability but also fall short in fully embodying the principles of sustainability and circular economy. To address this gap, we propose considering microbial fuel cells (MFCs) as a viable alternative and integral part of the renewable energy ecosystem. MFCs harness the omnipresence, abundance, and cost-effectiveness of their essential components, making them a promising candidate. Through our comprehensive analysis, we shed light on the limitations and advancements of this technology, which underscore the remarkable potential of MFCs to revolutionize our perception of clean, sustainable energy.

## Introduction

**Fig. 1:**
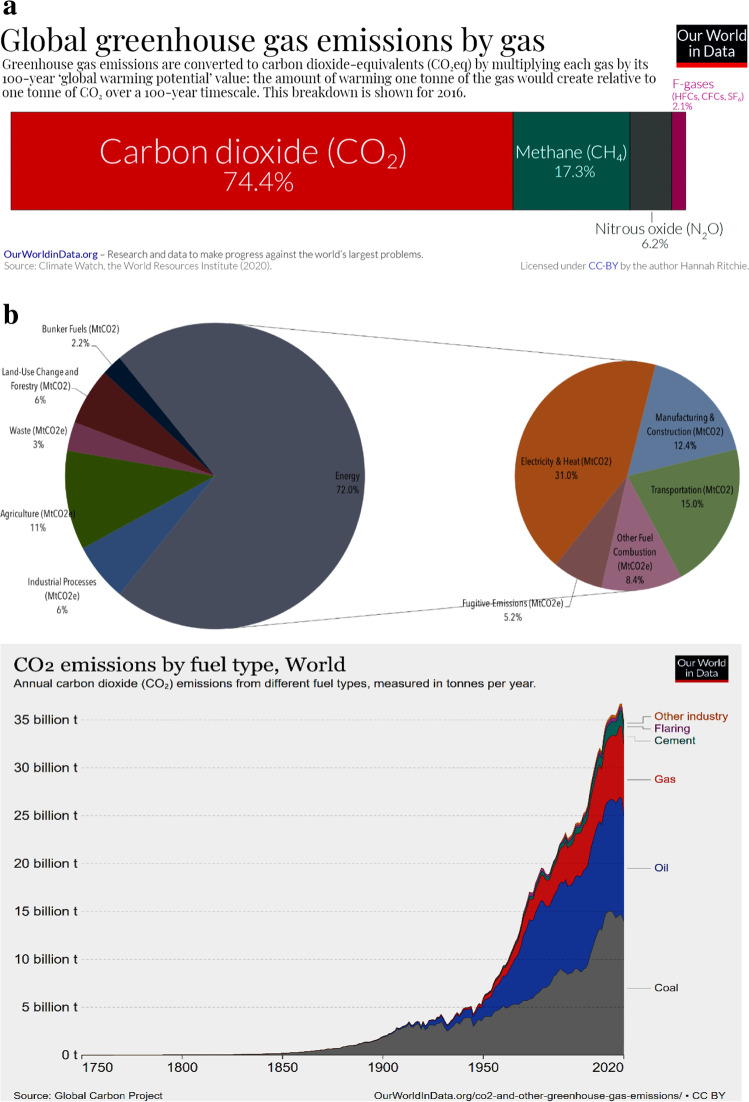
**a** Global inventory of GHG emissions. Adapted with slight modifications from Ritchie and Roser (2020). NB: Although N_2_O and F-gases make up a meagre fraction of the overall atmospheric remittance, they are considered more damaging than CO_2_. For example, N_2_O has a warming potential about 300 times that of CO_2_. **b** GHG (CO_2_) emission channels showing sectors and processes implicated (left pane) and the material or fuels involved (right pane). Adapted with slight modifications from World Resources Institute (2021) and Ritchie and Roser (2020)

Since the advent of the first industrial revolution (IR) and subsequent ones, the world has unconsciously embraced fossil fuels as a cornerstone of energy sources. This affinity stems from their versatility in industrial applications, particularly in the case of petroleum. This reliance has not only shaped geopolitical agenda for securing fossil fuel resources but has also had a profound impact on the cost of living and societal standards, driven by fiscal policies linked to the availability and distribution of these energy feedstocks. However, it is crucial to recognize that petrogenic resources are not only finite but may also be depleted earlier than anticipated. Furthermore, when these fuels are burned, they release a substantial amount of carbon dioxide (CO_2_), the most significant greenhouse gas (GHG) contributor (Fig. 1a) (Ritchie and Roser 2020). This alarming reality necessitates awareness, considering that nearly three quarters of global GHG emissions stem from the combustion of carbonaceous substances derived from fossil fuels (Fig. 1b).

The relentless emission of greenhouse gases (GHGs), predominantly driven by human industrial progress and daily activities, has led to a rise in global ambient air temperatures. This phenomenon has caused disruptions in weather patterns, energy flow, and nutrient cycling within ecosystems (Galloway et al. 2014; Unuofin 2020). Unresolved, the ongoing escalation of GHG emissions could lead to a significant increase in average global temperatures by at least 4 ºC by the twenty-second century (IPCC 2013), with potentially even greater impacts of 1.5 to 2 times that amount in equatorial and polar regions. To this end, the UN Climate Change Executive Secretary declared that the purpose of the COP27 conference was to actively implement the pledges and plans constituted at the previous climate change summits, especially limiting global warming potential to 1.5 ºC and reducing GHG emission by 43% by 2030 (UNFCCC 2022). The critical need to reduce our reliance on fossil fuels, coupled with the exponential growth of the global population and soaring energy demands, along with the imperative to decrease atmospheric GHG emissions, has necessitated a global shift towards alternative energy sources. These sources should prioritize characteristics such as renewability, environmental friendliness, sustainability, and cleanliness.

**Fig. 2:**
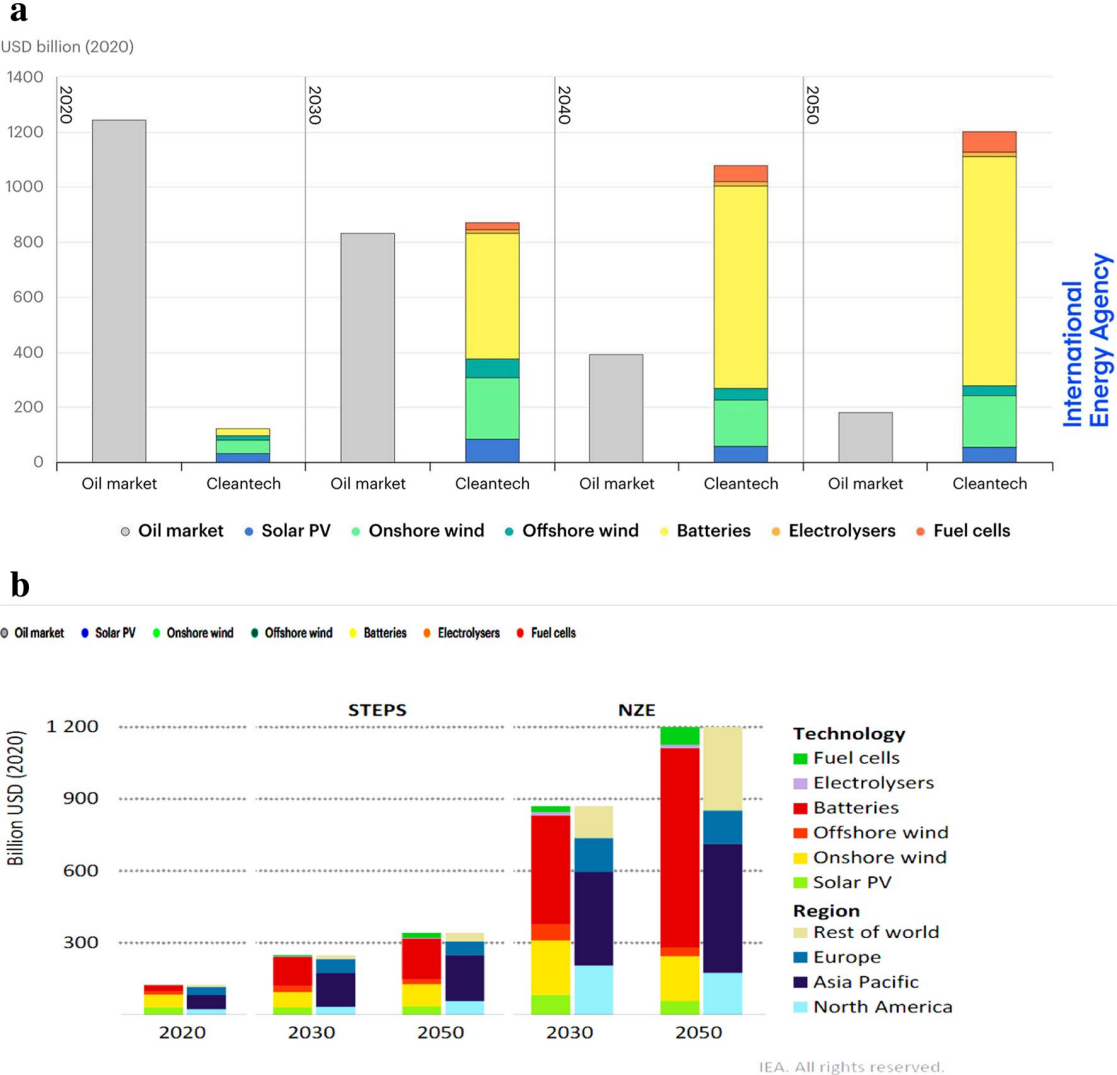
**a** Estimated market sizes of oil and selected clean energy technology equipment in the net zero scenario, 2020–2050. **b** Estimated market size for selected clean energy technologies by technology and region, 2020–2050

Renewable energy (RE) sources are widely recognized as clean alternatives derived from seemingly infinite natural resources and processes. Examples include solar energy from the sun’s radiation, wind, and hydroelectric energy from tidal movements and temperature changes. While fossil fuel–based energy sources are currently in high demand, they are projected to gradually be overtaken by clean technologies and materials as nations strive to achieve net zero emissions (NZE) by 2050 (Fig. 2a). This shift is expected to result in significant reductions in the market size and capitalization of fossil fuel–based energy (IEA 2021a). In line with the NZE mandate, annual investments in clean energy are forecasted to reach USD 4 trillion by 2030, surpassing the current developmental phases by more than triple (IEA 2021b). This will lead to job creation, with a focus on gender balance and addressing funding gaps between small- and medium-sized enterprises (SMEs) and established tech giants. However, it is important to note that while renewable energy sources are indeed renewable, their long-term sustainability is not guaranteed, as discussed further in the subsequent sections. Folklore-based beliefs about the sustainability of alternative energy systems can hinder the transition from fossil fuel consumption to a promising clean energy future. Among the various clean energy systems being deployed to reduce harmful emissions, fuel cells and batteries show steady progress in the market (Fig. 2 a and b). Fuel cells, in particular, hold promise for a sustainable future due to specific factors that will be explored in later sections.

Fuel cells are gaining rapid adoption in the developed world, finding applications in various sectors such as vehicle power, space exploration, and electric power grids. In the USA, for instance, approximately 260 MW of electric capacity was generated by 166 operating fuel cell–based power generators in 113 facilities as of the last quarter of 2021 (US Energy Information Administration 2022). Despite this progress, the US Department of Energy (DOE) recognizes the need to address technical barriers related to fuel cell development, particularly cost, performance, and durability (energy.gov 2022). In the pursuit of clean and renewable energy, one type of fuel cell, known as microbial fuel cell (MFC), has been largely overlooked by both government and industry participants. The challenges associated with scaling up and commercializing MFCs have led to the perception that they are not economically or technologically viable. Moreover, it is worth noting that MFCs offer unique sustainability advantages that few other renewable energy systems can provide. However, to our knowledge, there is paucity of critical discussions and appraisal of the sustainability of emerging energy technologies as well as the prospects of MFCs in bridging the gap between sustainability and a circular bio-enabled economy. Therefore, this review aims to achieve the following objectives: (i) analyze and distinguish the concepts of emerging energy technologies in context of their implications for the environment and socio economic obligations; (ii) enumerate prominent new generation energy systems, highlight their perceived impacts and issues involved with their commissioning/decommissioning, as well as critically discuss common sustainability barriers attributed to them; (iii) reveal the significant role that MFCs can play in the nexus of renewable energy, sustainability, and the circular bio-enabled economy.

## The major energy conundrum: being renewable yet sustainable

**Fig. 3:**
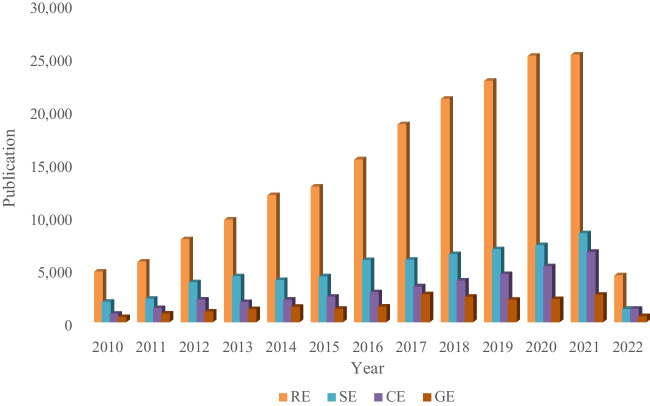
Number of publications with themes related to renewable energy (RE), sustainable energy (SE), clean energy (CE), and green energy (GE) in the last decade (accessed April 20, 2022)

The desire for a clean, renewable, and sustainable environment has been present among world leaders since the mid-twentieth century. However, significant efforts to address environmental and energy crises were only enacted later in the same century. Today, in the twenty-first century, resolving these crises has become a top priority globally, prompting nations to undertake the transition to renewable and sustainable energy through incentives and joint efforts. Since the signing of the Paris Climate Treaty in 2016, research has focused on understanding various environmentally friendly energy concepts: renewable energy (RE), green energy (GE), sustainable energy (SE), and clean energy (CE). While these terms are often used interchangeably, they convey different ideas. In this review, we will distinguish between these four important aspects of achieving a sustainable environment. GE refers to sources or technologies that emit reduced amounts of pollution, such as greenhouse gases, radiation, or chemical contaminants, to ensure they do not harm the flora, fauna, or microbiota of a specific ecosystem. On the other hand, CE goes further by implying sources or technologies that emit negligible amounts of pollution, having minimal to no impact on the environment. RE refers to energy sources that can be replenished within a relatively short period (e.g., daily, biannually, or biennially) and should ideally meet energy needs before their regenerated forms become critically required. SE, on the other hand, involves resources and technologies that can meet current energy needs without compromising the ability to meet future demands, ensuring environmental and socioeconomic obligations are not compromised. Considering the challenge of achieving CE in the present era, GE can pragmatically serve as the umbrella term for desired energy systems, as it emphasizes the reduction of emissions. Data from the Web of Science (WoS), accessed in April 2022, indicates that RE (208,542 outputs) and SE (65,558 outputs) were the most researched topics compared to GE (20,309) and CE (38,857). This trend has remained consistent over the past decade, with GE being rarely explored (Fig. 3). Interestingly, research on GE and CE has been led by institutes in the Eastern Hemisphere, while RE and SE have been dominated by institutes in the Western Hemisphere. Although the exact reasons for this are unclear, it is possible that regional convenience and guidance from energy experts and policymakers influenced the choice of these themes to convey urgent and relatable messages to their respective populations. To accelerate the transition away from fossil fuels, President Obama referred to emerging energy sources and technologies as renewable and clean interchangeably (Obama 2017). However, debates persist on which sources should be classified as both renewable and sustainable. Sustainability is crucial for a harmonious future for the Earth and its inhabitants. Energy sources or systems aiming to achieve sustainability must be strategically utilized in alignment with political, environmental, social, and economic factors (Yoro et al. 2021). The energy return on investment (EROI) is a critical metric, reflecting the useful energy produced compared to the energy invested in assembly, maintenance, and decommissioning (Kelly 2016). A low EROI coefficient (≤ 10) indicates a project with limited benefits beyond technical feasibility, often considered immature or exploratory. Many emerging energy systems struggle for relevance and seek widespread adoption, facing scrutiny due to their sustainability shortcomings. Achieving exclusive and comprehensive *renewable yet sustainable* energy remains an unresolved challenge in the industry. Therefore, researchers, policymakers, and industry stakeholders should exercise caution in declaring certain energy systems as *renewable yet sustainable*.

## A well-balanced succinct appraisal of some existing new generation energy systems

There have been ongoing debates and discussions about the definition, classification, relevance, feasibility, and integration of modern energy systems. Various researchers have expressed their opinions and counterarguments on these topics (Heard et al. 2017; Brown et al. 2018; Harjanne and Korhonen 2019; Steffen and Patt 2022; Adebayo et al. 2023). While it is widely agreed that contemporary energy systems are necessary to impede the accelerated occurrences of global warming and potentially restore climate-impacted communities, there are a number of sustainability issues (both upstream and downstream) involved with the initiation, performance and post-operational management of these systems. Therefore, this section aims to deliver an unprejudiced assessment of some of these systems, which would serve as a roadmap for emerging energy initiatives and already extant, fully operational systems, in order to ensure their cognizance and compliance of the sustainability concept.

### Wind energy

Wind movement plays a critical role in the aerodynamics of turbines and their ability to generate electric energy from mechanical energy. Besides the potential devastating effects of downdrafts and downbursts, harnessing wind power has significant benefits. It is a widely available natural resource that can contribute to climate change mitigation and serve as a source of livelihood and revenue. Currently, wind energy is one of the leading clean technologies in the global market, expected to experience substantial growth by 2030 (Fig. 2a) (IEA 2021a). Installation rates and capacity generation have increased both regionally and globally (Leite et al. 2022; Singh et al. 2022; Verma et al. 2022). However, offshore wind coupling lags behind onshore facilities, likely due to specific requirements for critical raw materials (CRMs) (Li et al. 2022a). The assembly and operation of wind energy systems may have various implications, which may include:

(i) *Increased land use (except in hilly terrain)* — This is mostly associated with onshore systems. On a level ground, more spacing might be needed between each erection; artificial elevations would require more raw materials and hence cost-intensive. Conversely, due to larger structure of offshore turbines, installation and operation might interfere with customary anthropogenic and ecological activities (Bjärstig et al. 2022).
(ii) *Threats to wildlife and habitat* — There might be erratic occurrences of avian deaths, resulting from collisions with high-speed blades. During heavy winds, birds might be swayed towards the direction of the blades; moreover, suction pressure that might arise from the centripetal force of the spinning blades might pull the flocking birds towards a disastrous fate (Conkling et al. 2022). However, the National Wind Coordinating Committee (NWCC) advised that such occurrences are too inconsistent and inconsequential to pose a serious threat to species populations.
(iii) *Visual and sonic nuisance* — Operation of wind energy systems usually accrue suburban wellbeing concerns, especially from sonic disruption of an otherwise serene environment. This partly stems from the air resistance as the turbine blades beat through the air; the other reason might be the mechanical sound associated with the operation of the turbine’s generator. Altogether, the level of sonic pollution might be attributed to the operating speed as well the turbine design, composite materials of the blade, its surface area, and also the tribological occurrence in the system. Although, no direct adverse impacts have been reported, with regard to human health, the nuisance might indirectly affect sleeping patterns of residents nearby and particularly impact vital communication among wildlife (Conkling et al. 2022; Teff-Seker et al. 2022). Meanwhile, the rotational movement of wind turbine blades in sunlight or daylight reflects the sun, creating light and shadow flickers which might compete with visual concentration of motorists (Msigwa et al. 2022). Based on these concerns, attitude towards adoption of wind energy as a renewable energy source in some areas is quite lethargic or negative (Cranmer et al. 2020); correspondingly, there has been a drastic drop in real estate values by 5.4% (Dröes and Koster 2021).
(iv) *Life cycle–associated issues* — The life span of a wind turbine is estimated at 25 years; therefore, an appraisal of certain factors regarding its installation, and maintenance during its maiden performance is critical to discerning its appositeness for adoption as a clean technology. This will include economic, environmental, and socio-political issues that are involved with critical raw materials prospecting, production and transportation, composites smelting and moulding, on-site construction and assemblage, operation and maintenance, demounting and decommissioning, and ultimately recycling or dumping. From environmental purview, wind turbines are asserted to impose heavy footprints, as they are regarded as ambassadors of fossil fuels. This is because numerous stages of their lifecycle are intrinsically influenced by petrogenic and non-petrogenic fossil fuels (Smil 2016; Emblemsvåg 2022); hence, the larger the capacity of electricity output intended from the system, the higher the patronization of fossil fuels, which already derides its role in environmental greening.

### Solar energy

The energy harnessed from the sun is incredibly powerful, as an hour of maximum radiation can generate more than enough energy to meet the global energy consumption for an entire year, according to the US Department of Energy. In theory, if we had optimal technologies in place to harness, convert, and distribute this energy, just a few hours of sunlight could fulfil the world’s annual energy demands. Over the past decade, solar energy has experienced significant growth, becoming the fastest-growing source of energy and accounting for approximately half of all investments in renewable energy (Frankfurt School-UNEP 2020). This expansion has not only created numerous jobs and wealth but has also seen a decline in costs per solar module, indicating an inverse relationship between technological advancement and expenses, as noted by Kabir et al. in 2018. Solar energy systems have played a crucial role in reducing carbon emissions, as exemplified in California, where the installation of 113,533 household solar systems has led to the avoidance or reduction of approximately 696,544 metric tons of CO_2_ emissions (Arif 2013). However, the environmental impact of solar energy systems can vary depending on their scale, ranging from small, distributed rooftop photovoltaic panel (PV) arrays to large utility-scale PV and CSP (concentrating solar thermal plants). Like any other energy system, it is important to consider potential impacts, which may include the following:

i) *Glaring, singeing, and flaring* — Just like wind turbines, CSP and PV might pose a threat to certain migratory birds and also insects. Due to the concentration and corresponding reflection of intense sunlight, feathers of migratory birds might be burned or singed, while insects might be incinerated whole, hence reducing their species robustness (Chock et al. 2021). Moreover, glares from the erected structures might present ocular health issues or impairment in animals or discomfort in humans, thereby impacting the social behaviour of resident animals (Chock et al. 2021). Critically speaking, poorly synthesized cells and unintelligently coupled and poorly sited solar energy systems might incidentally contribute to wildfires, as they have been linked to house fires. Damages to property have been recorded in different events in the USA, the Netherlands, and Germany, respectively (Business Counselor Office in the Netherlands 2013; Sipe 2016). Consequently, these fires not only reduce the capacity of electricity generated but also emit toxic gases and hydrogens compounds, such as HF and HCl (Wu et al. 2020).
ii) *Land use and degradation* — The solar energy harvested by solar energy systems is directly proportional to the size and number of modules present, which usually requires extensive areas of land depending on solar intensity, geographical relief, and technology deployed: hypothetically, 3.5 to 10 acres per megawatt output of utility-scale PV and 4 to 16.5 acres per megawatt of CSP installations. In this regard, solar energy systems are the most land intensive renewable energy modules in terms of energy trade-off, and agricultural prioritization of land resources implies a reduction in energy harvested as corroborated by previous studies (Dias et al. 2019; Pearlmutter et al. 2020). Thus, installation of solar energy might compete heavily with other land-based activities and entities. For example, the fast pace of solar systems advancement in the USA has been prognosticated to acquire thousands of acres (Cagle et al. 2019). Solar energy structures might likewise serve as agents of land deterioration. This is because they may prompt localized erosions arising from consistent trails of concentrated stormwater fluxes that trickle or wash off solar panel surfaces during snowmelts or downpour. Ultimately, the soil profile will be affected as well as its nutrients and organic content, thereby spearheading the sparse distribution of vegetation cover, impacting local biodiversity (Rabaia et al. 2021), and also creating extensive water body temporary embankment due to wash down of sediments.
iii) *Water footprint* — Although it is generally believed that solar energy systems are lenient in water demands, their water requirements indeed differ at successive stages of their life cycle, from structure erection to demounting. Moreover, as required in major manufacturing processes, water is used to manufacture solar PV components; hence, wastewater treatment and reusability should be integrated in their workflow of production. Water expenditure depends on the plant design, plant location, and the type of cooling system (Nathan et al. 2015; Dhar et al. 2020). CSPs, like all thermal electric plants, require water for cooling. For example, CSP that hybrid (wet-dry) technology with cooling towers withdraw between 600 and 1300 L of water per megawatt-hour (L/MWh) of electricity, which is not as effective as the wet cooling technology that consumes 3100–3800 L/MWh (Ziapour et al. 2017; Saria et al. 2018). Interestingly, antithetical to earlier assertions, PV require some water quotients for their cooling, as a phenomenal increase in their systems temperature (above room temperature) will plummet its efficiency and performance (Rabaia et al. 2021). Aside from cooling requirements, about 20–150 L/MWh water withdrawals are also essential for mirrors and panels cleaning in order to maintain optimum reception of solar radiation (Nathan et al. 2015); however, water withdrawal quotients and cleaning frequency will depend on biotic, abiotic, and technological factors (Saria et al. 2018). Water requirements would pose a threat to most desperate water needs, especially in regions renown for water scarcity and/or water stress.
iv) *Hazardous materials* — PV cell manufacturing processes adopt a number of hazardous materials, especially during solar cells extraction or etching and cleaning the semiconductor surface (Marwede et al. 2013; Üçtuğ and Azapagic 2018). These materials and chemicals include hydrogen, hydrochloric acid, sulfuric acid, nitric acid, hydrogen fluoride, ammonia, selenium hydride, silicon, and isopropanol, which are corrosive, flammable, and carcinogenic (Tawalbeh et al. 2021). The amount and type of chemicals used depends on the type of cell, the amount of cleaning that is needed, and the size of silicon wafer. Correspondingly, emissions of hazardous gases and chemicals would depend on the type of PV cell materials (Aman et al. 2015). For example, thin-film PV cells comprise and emit more toxic materials than those used in traditional silicon photovoltaic cells, particularly copper-indium-gallium-diselenide, gallium arsenide and cadmium-telluride. Since these materials represent both critical resources and agents of adverse environmental and public health, they should be recycled at end-of-life of the solar module.
v) *Life cycle–associated issues* — Similar to the wind turbine, the solar panel is most active for 25 to 30 years; however, suboptimal performances might still be afforded thereafter. As observed with wind turbines, the production of PV cells is still heavily invested in non-renewable fossil fuel sources; about 73.90% is demanded therein (Vácha et al. 2021), albeit having a strikingly low energy performance when compared to non-renewable fossil fuels or even renewable biomass and hydropower. Moreover, its environmental impacts have been immensely felt, primarily on human health (35.00%) and then climate change (19.90%), resources (19.01%) and ecosystem quality (4.68%) (Vácha et al. 2021). Resources used are attributed to not only the panels but also the battery systems and inverters used to store excess solar energy as chemical energy and DC to AC conversions, respectively. Worthy of mention, the scramble for locally distributed critical resources has been observed to impose socio-political impacts, which will be summarily discussed later.

### Geothermal energy

Geothermal energy has significant potential to make a substantial contribution to the global renewable energy market. The Earth’s inner core contains an estimated 42 million megawatts of thermal energy that can be conducted to the surface. However, only a small portion of this energy has been tapped for electricity generation worldwide. Currently, approximately one tenth of countries around the world are generating a total of 73.7 terawatt-hours (TWh) per year from geothermal sources. It is projected that increased adoption of geothermal energy will result in the production of 800–1300 TWh of electrical energy and 3300–3800 TWh/year of direct thermal usage in the next two decades (van der Zwaan and Dalla 2019; Coro and Trumpy 2020). Geothermal power plants primarily rely on pressurized hot fluids or steam extracted from localized excavations in the Earth’s crust, making them less vulnerable to fluctuations in fossil fuel prices or geopolitical conflicts. However, the low adoption of geothermal energy compared to other renewable sources is due to various challenges encountered throughout the development and decommissioning processes. In addition to unproductive wells that result in wasted human and capital investments, these processes can also have significant environmental consequences. The environmental implications of geothermal plants depend on the method used to derive electricity (direct steam, flash, or binary) and the cooling technology employed (water-cooled or air-cooled). Despite these considerations, there are critical issues that hinder the widespread adoption of geothermal energy systems. These issues include:

i) *Water quality issues* — The water footprint of geothermal power plants is not inconsequential; depending on the cooling technology adopted, about 722 gallons of water per MWh may be required (Mekonnen et al. 2015). This could interrupt freshwater availability and distribution to attend basic needs, if freshwater is solely used in cooling. However, most extant facilities operate closed-loop water systems, where the water extracted is backwashed into the geothermal reservoir after it has been used in generating electricity or heat. Moreover, hybrid systems can use either freshwater or geothermal fluids for cooling, where the latter would theoretically alleviate the water footprint. Notwithstanding, water quality remains a disturbing issue; hot water pumped from underground reservoirs (usually in steel well casings) often contains high levels of sulfur, salt, and other toxic minerals, above environmental standards (Baysal and Gunduz 2016; Li et al. 2022b), which might over time corrode the casings and then seep through to contaminated pristine groundwater resources. Correspondingly, these toxic chemicals are present in wastewater discharged to nearby aquatic milieu, thereby adversely impacting florae and faunae, and ultimately human health.
ii) *Greenhouse gas emissions* — Apart from the abundance of gas in the atmosphere, there is also considerable amount of pressurized gases trapped in the lithosphere, which are released from the well during deep drilling activities as blowouts. As such, it has been revealed through research that wastes of geothermal plants, especially open-loop systems, contain a cocktail of greenhouse gases, such as CO_2_, CH_4_, H_2_S, and NH_3_, *inter alia* (Finster et al. 2015; Karapekmez and Dincer 2021). In a closed-loop approach, atmospheric emissions are controlled by injecting the gases back into the ground after giving up their heat. The bulk of GHGs emitted is H_2_S, which has a distinctive effluvium, and is transformed into SO_2_, once retained in the atmosphere (Choudhary et al. 2021). The contribution of SO_2_ to acidification of soils, crops, vegetation, and natural water bodies cannot be neglected; moreover, its role in formation of acidic particulates that are absorbed by humans and thereafter effect heart and lung complications is worth of note. Traces of mercury emissions have been observed likewise (Tut Haklidir and Haklidir 2020), whose exposure can trigger neurological and behavioural disorders, or even death in cases of high exposure.
iii) *Land use and inequities* — Land requirements usually depend on diverse factors; some critical ones include the following: the geomechanical/geotechnical properties of the resource reservoir, the type of energy conversion system, energy capacity, the type of cooling system, and the substation and auxiliary building needs. Typically, about 1–8 acres of land is mostly allocated per MW of energy; oftentimes, this minimal land requirements permit their setup in protected locations and farmlands (Bravi and Basosi 2014). However, exploiting geothermal resources close to human settlement and sources of livelihood as well as vegetation has been shown to cause harm to flora and faunae, land degradation through seismic events, as well as marginalization and forceful relocation of natural inhabitants due to project expansion. Anecdotes of such occurrences have been thoughtfully communicated by Kabeyi and Olanrewaju (2022); correspondingly, a SWOT (strengths, weaknesses, opportunities and threats) analysis corroborates the concerns we raised (Rahman et al. 2022). With particular regard to seismic events, the siting of geothermal systems on geological “hot spots,” with high earthquake tendency might increase earthquake occurrence, especially if the process employed is similar to hydraulic fracturing observed for natural gas entrapped hot rocks. As a preventive measure, geothermal plants should be sited at an appropriate distance away from major fault lines and also from densely populated areas. In cases where the latter is unachievable, constant monitoring and transparent, equitable communication with local communities must be ensured.
iv) *Life cycle–associated issues* — In open-loop geothermal systems, about 10% of air emissions are CO_2_ and a smaller amount of emissions are methane, a more potent global warming gas. Geothermal plants contribute approximately 380–1045 kg of CO_2_ eq/MWh to the global warming inventory, which is not dissimilar from gas- and coal-based power plants (640–1068 kg CO_2_ eq/MWh) (Bravi and Basosi 2014). Fossil fuels are also invested in the drilling, construction and pumping processes, which are the phases that consume petrogenic fuels most. Successive evaluation of life cycle indicators by different researchers has shown that resource consumption, eutrophication, land exploitation, human health impacts, and global warming are major unfavourable feedbacks associated with the execution and decommissioning of geothermal power projects (Tomasini-Montenegro et al. 2017; Wang et al. 2020; Zuffi et al. 2022).

### Biomass energy

Biomass is not only our oldest energy source ever used, but also the most heterogenous, flexible, and ubiquitous form of energy. This is because it is currently imbued with an inexhaustible cache of upstream resources and growing downstream utilities. In this regard, its feedstock resources are structurally, geographically, and ecologically diverse, yet ubiquitous, such as energy crops (like switch grass), agro-industrial residue, animal and human excrement, municipal sludge, and even bryophytes and algae. In the same vein, based on treatment processes involved, feedstock may be converted into solid fuels or used to synthesize liquid fuels that are ultimately combusted to produce heat energy and subsequently electricity. In 2021, biomass contributed about 4.835 trillion British thermal units (TBtu), which is comprised about 5% of total primary energy consumption in the USA; interestingly, about 2.313 TBtu (48%) and 1.477 TBtu (31%) of total biomass energy were diverted to meet industrial and transportation needs (EIA 2022). However, about 10% and 9% were utilized residential and electric power purposes, which is expected to grow in the not-too-distant future. Moreover, solid fuels, in form of wood pellets and other densified biomass fuels have produced to commercial and export scale; about 8 million tons of wood pellets were exported by the USA in 2021 (EIA 2022). Similarly, the potential of solid biomass for heat energy and electricity has been expressed in certain developed countries. For example, in Denmark, Estonia, and Finland, at least 15% electricity is generated through combined heat and power from biomass resources. Liquid biofuels have also derived 15% adoption, replacing fossil fuels used in heating and transport in Brazil and Sweden (IEA 2021c). Moreover, biomass energy is important, so much so that certain countries with constrained indigenous forest biomass resources (Denmark, the UK, Belgium, and the Netherlands) outsource their supply of solid biomass (IEA 2021c). However, the environmental impacts would be determined by the type of feedstock and the manner in which it is developed and harvested. In this regard, anticipated issues are outlined hereunder:

i) *Water use and abuse* — Depending on their technology, average water apportionments for the cooling of biomass-powered energy systems are not dissimilar coal and nuclear power plants requirements. In particular, in biomass plants with once-through cooling systems, which circulates water from nearby sources before discharge as used water, roughly 20,000–50,000 gallons (75,708–189,271 L) are invested per MWh (Dilthey 2018), with an irrecoverable consumption of 300 gallons per MWh. In most cases, used water is returned warmer and more polluted to its source of abstraction, which often has a negative impact on plant and animal life. Other issues pertaining to water use for biomass would include the degradation of regional water quality, due to runoff from acceleratingly cultivated energy crops (usually requiring more water than regular industrial and domestic needs) using chemical fertilizers.
ii) *Land use and abuse* — Since the relaxation of competition between end uses of edible crops, for human or energy, through the introduction of 2nd generation biofuels, edible crops have plateaued in costs per unit. However, substantive production of energy crops would require massive land mass; this would definitely encroach into conserved natural ecosystems such as forests and jungles, thereby threatening and displacing wildlife and also waning the natural fortresses that stymie the acceleration of global warming. To put this in perspective, IEA (2021d) predicted that the total land area earmarked for bioenergy production a realizable NZE scenario would increase by 80 million hectares (Mha) by 2050. Of the increase, 30 Mha are new forests, which represent no significant increase from present day global forest area, whereas 50 Mha would constitute land for short-rotation woody crops and energy crops. Moreover, the continuous tilling and harvesting (which is observed for a few energy crops) would adversely affect soil profile and chemistry, which could present as localized erosions (in areas with much reduced vegetation cover) as well as dramatic loss of soil nutrients, microfuana, and microbiota.
iii) *Air emissions* — Biomass combustion for electricity generation is not beneficial to air quality, overall; however, emission intensity usually depends on the combustion systems, feedstock used, as well as pollution control schemes integrated in the process. Aside from aerosols, biomass combustions may exude CH_4_, NOx, N_2_O, CO, and SO_2_. While N_2_O and CH_4_ are guarantors of tropospheric ozone formation (especially the former), which mimics GHGs; CO and SO_2_ are notorious enforcers of respiratory discomforts (Tomlin 2021). Although, biomass facilities usually emit less SO_2_ and mercury than coal, their NOx emissions are higher than natural gas; even so, both gases (SO_2_ and NOx) could facilitate acid rain and formations of toxic particulate matter.
iv) *Life cycle–associated issues* — Unlike in other systems, there is no one-size-fits-all approach to accurately account for life cycle assessment of the blanket biomass energy system. This is because of the labyrinthian network it exhibits, from upstream to downstream. In this regard, environmental and socioeconomic footprints would be determined by the pathway a particular biomass energy system follows, from cradle to grave. Audit of plant biomass energy systems reveals global warming emissions attributable to growing and harvesting biomass feedstock, transport, and burning or gasifying the feedstock. Although metrics for transportation and combustion emissions are approximately parallel for all types of biomasses, emissions from the sourcing of biomass feedstock vary expansively. On account of popular consensus, biomass presumably had net zero global warming emissions, due to carbon balancing exhibited by biomass growth and subsequent combustion. However, carbon balancing might only be applicable in appropriately monitored and regulated systems. In scenarios where plant matter is diverted to landfill, there is disruption in the assessment of that particular process, because the waste biomass becomes part of a cocktail that collectively decomposes to release methane, a potent global warming gas. Thus, diverting these wastes for electricity production not only reduces landfill volume methane emissions, but also helps in the effective audit of the biomass system appraised. In corroboration, Xu et al. (2021) claimed that screening bioenergy projects on specific feedstock types alone is insufficient, as emissions might depend on a lot of intrinsic and extrinsic attributes.

### Water-based energy

This category encompasses energy systems that are designed to harness and convert certain energetic properties of certain water bodies ultimately into electrical energy. In this regard, well-known examples of these systems include hydroelectric energy, tidal energy, ocean energy, and osmotic energy; however, hydropower is the most mature, in terms of project execution and development. Hydroelectric power is harnessed when potential energy of high-altitude water bodies (mostly dammed) is released by gravity to facilitate production of mechanical energy from kinetic energy of a spun turbine, which is ultimately converted to electrical power. Hydropower is a well-studied and utilized form of energy as it individually generates 17% of global electrical energy supply (IRENA 2020) and has the highest installed capacity among all renewable energy systems (Renewables 2020). Notwithstanding, other water-based energy systems are gradually growing with immense potential to fill up the hiatus in electrical energy supply; a concise account of such possibilities has been given by Rahman et al. (2022). An optimistic outlook would enunciate water-based energy systems as the closest we can get to equitable electrical energy supply, since water resources are cosmopolitan; on the other hand, an accelerated and unregulated move toward commissioning water-based energy project might play out as a “Greek gift” due to the following issues:

i) *Land use and abuse* — This environmental issue is peculiar to hydroelectric power systems. Reservoir size determines the expanse of land that must be relinquished to a hydroelectric project; however, this may vary widely, depending largely on the size of the hydroelectric generators and the topography of the land. The geographical relief of gorges is advantageous in that it natural provides great depths (reservoir) for phenomenal volumes of water. However, level lands would require extensive landscape for water flooding, which will encroach human settlements (often creating sociopolitical conflicts), wildlife habitats, as well as sacred landmarks. For example, the Balbina hydroelectric power project invested about 2360 km^2^ landmass to generate 250 MW of power; meanwhile, 0.253 km^2^ is required for a 10-MW power plant (Fearnside 1989). Moreover, some catastrophic events, such as reservoir-induced seismicity, resulting massive mud slides and earthquakes: events traced to the Gorges dam in China (2003–2020) and the Brumadinho Dam in Brazile (2019) do not make it a sustainable option for power generation.
ii) *Aquatic ecosystem entropy* — In general, the installation and operation of water-based energy systems, in the long run, instigate various degrees of entropy in the ecosystem. Typical perturbations experienced include turbine injuries sustained by aquatic life and wildlife, increased sedimentation, nutrient, chemical and toxic metal leakage and accumulation, algal bloom and eutrophication, habitat fragmentation that causes changes in migratory patterns of fishes and wildlife (Palmeirim and Gibson 2021; Baird et al. 2021), constrained reproduction in certain fish species, as well as exposure of other aquatic fauna to intense predation. In some cases, considerable amounts of CO_2_ and CH_4_ emissions might occur alongside the decline in dissolved oxygen. Ultimately, this entropy affects human socio-economic development, especially for those derive livelihoods from fishing activities or freight and also rendering unsafe scarce water resources meant for human consumption.
iii) *Life cycle–associated issues* — Similar to biomass energy systems, pinpointing life cycle assessment of water-based energy systems with a blanket approach is either cumbersome or unachievable, due to the marked differences in their material requirements and structural designs. Unlike most other renewable energy technologies (20–30 life span), water-based energy technologies, particular hydroelectric energy systems, are effectively operational for at least 100 years. This long operating lifetime has been ratiocinated to offset indirect emissions and other impacts that would have arisen during manufacture, transportation, and coupling of key equipment and building structures (EIA 2021). However, after critical evaluation of diverse cases studies, an extensive width of 1.5–3747.8 g CO_2_ eq per kWh was realised, whose variability was characteristic of intrinsic and extrinsic factors (Gemechu and Kumar 2022). Although the bulk of emissions has been attributed to the construction phase of certain systems (Chu et al. 2022) as well as the choice of materials used (Aldawoud et al. 2022), an extensive review of literature posits that not less than 90% emissions may be attributable to reservoir GHGs (Gemechu and Kumar 2022). Aside from GHG emissions, an evaluation of a battery of micro-hydropower installations has also revealed the acidification, human toxicity abiotic resource depletion potentials, which are worthy of note (Ueda et al. 2019).

## Confocal discussion of sustainability barriers

We have earlier hinted what criteria contemporary energy systems must fulfil in order to accede sustainability (*supra*); however, the path thereof is riddled with hurdles that must be overcome. In addition to the issues succinctly enumerated in the preceding sections, we hereby give a confocal perspective to other underlying issues. Among them, intermittency is considered a strong barrier, which has been constantly used by fossil fuel apologists and lobbyists to dissuade major stakeholders against venturing into alternative energy technologies. In the true sense of the word, certain energy systems are incapacitated by the inconstance of energy drivers; solar, wind, and tidal energy systems are particular impacted in this regard. This is due to the perpetually irregular or seasonal availability of solar radiation and air currents, where optimal energy output is only guaranteed during peak periods. Moreover, their integration into electrical grids (usually supported by conventional power systems) could bring along with it the unexpected changes or imbalances in voltage production and consumption and random erratic yet continuous interruptions on short- and long-term operations (Notton et al. 2018). As such, efforts to balance out intermittency include accurate forecasting of peak periods on variable energy sources, which is cumbersome or smoothing variability effects by energy storage, which comes with additional costs (Gowrisankaran et al. 2016; Notton et al. 2018). Syntheses of literature on impacts of integrating intermittent renewable energy systems (IRES) into conventional power grids have been provided by Mlilo et al. (2021) and Asiaban et al. (2021), respectively, where the former discussed the technical challenge intermittence could confer on the confer on the power systems security, flexibility, strength, stability, and reliability. We observed that there were corresponding opinions between the two research teams; however, Asiaban et al. (2021) further hinted that the integration of IRES into the conventional power grid might in the long run cause disruptions due to consistent load variability, leading non-optimal performance of the power plant. This reduced efficiency of power plants would among other things increase their fuel consumption and, unsurprisingly, CO_2_ emissions. Conversely, increased installation of IRES might accrue negative prices to the commodity for balancing, in terms of current redispatch to reduce local grid overload, and also voltage redispatch to regulate local over- or undervoltages. In this regard, some EU countries, especially Germany have experienced, first hand, the remission of taxpayer’s money for regulation of excesses: In 2016, 97 h excess in electricity production, resulting in a 3.83-TWh surplus accrued an extra cost of 70 million Euro for export costs. It was also observed that close to 1 billion Euro might be devoted to redispatch interventions (Ongena et al. 2017). Moreover, taxpayer’s money still serviced the installation of phase shift transformers, beyond German borders, especially in Poland and Czech Republic, in order to reflect back cataclysmically high surpluses of German electrical energy exports, when their respective national grids are overloaded (Boldiš 2013). While the energy excesses of IRES could be repurposed for heat supply and mobility (often requiring huge capacity), storage becomes imperative to curtail excesses, both in production and losses. However, bearing in mind the evanescence of electricity (which necessitates its transformation into other energy forms for conservation), storage efficiency would be determined by techniques and technologies employed. It should be considered also that choice of techniques and technologies would be determined by the capacity and immediacy of the energy produced. For instance, a fast response yet low energy capacity is observed for electrochemical energy storage (batteries), whereas mechanical and thermal energy storage might undergo some time-lagged transitions before eventually yielding electrical energy, despite their large and long-term storage capacity. However, cost implications using different storage modules, seasonally or annually, suggest the use of non IRES with more mature commissioning (biomass), which could cut back 20% of electricity costs (Sanchez et al. 2022). Overall, these techniques and technologies have not yet outrun their respective peculiarities; moreover, if all storage capacities assembled worldwide so far were combined (512 GWh), it would only suffice for 10 min of steady electric power (Ongena et al. 2017). Notwithstanding, there is growing research interest to improve thereupon, particularly the synthesis of smart and portable composites capable of long-term high-capacity energy storage.

**Fig. 4:**
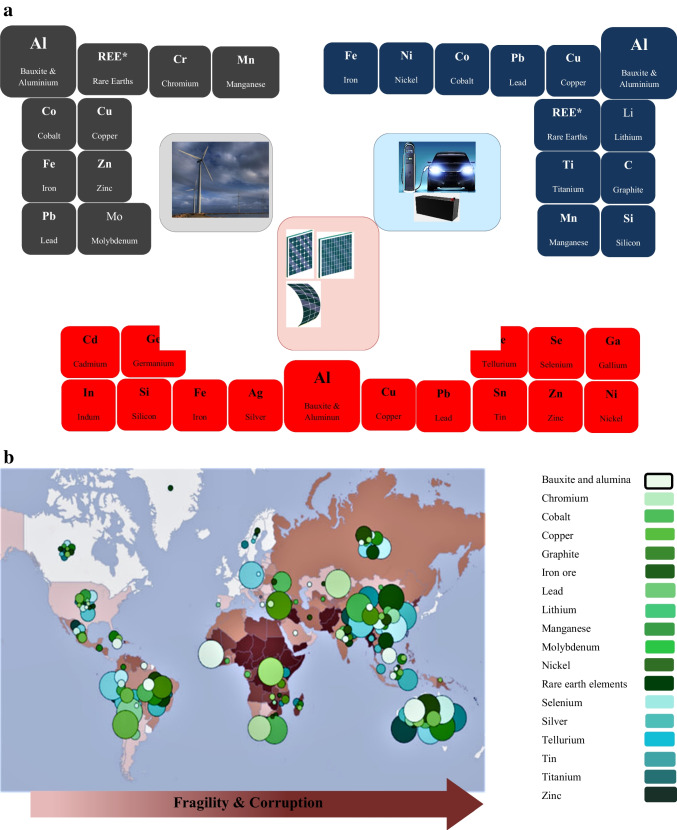
**a** Periodic representation of critical raw materials of relevance in wind energy (gray), solar energy (red) as well battery systems (blue). **b** Global reserves of critical raw materials for green energy technologies and corresponding intensity of fragility and corruption measures: instruments of crises. Mineral resources are colour-coded accorded the legend and intensity of fragility and corruption is directly proportional to colour intensity. Australia shares the same shade of colour as Canada, Greenland and Scandinavia (adapted from Church and Crawford (2018) with modifications). Source: Fund for Peace (2018), Transparency International (2017), U.S. Geological Survey (2018)

More than ever, the desperate propulsion towards RES has led to incidences of resource criticality, where certain raw materials that are sine qua non for the manufacture of RES scaffolds and parts, and pivotal to the socio-economic sustenance of the importing and exporting communities encounter risks in supply flow and are subsequently rendered vulnerable due to overdependence of their systems and economies. In other words, resource criticality is a scenario where majority of desperately demanded resources, worldwide, are only produced or supplied by a few countries, which renders dependent countries vulnerable in times of geopolitical conflicts: A first-hand experience witnessed today is Russian oil and gas vs the EU27. In this regard, resources, often denominated “critical raw materials” (CRMs) inexorably influence the planning execution and decommission of certain RES projects, especially solar- and wind-actuated as well as storage technologies (Fig. 4a).

From a perspective given by Mancini et al. (2018), we were able to identify major drivers of resource criticality and also how to regulate resource flow in harmony with different degrees of human needs; however, we would discuss further on the major disruptor of idea of sustainability (as defined *supra*): conflict. The saying *more money more problems* has not only aged well but is overwhelmingly relatable to abundance of CRM reserves in a politically unstable society. If done transparently and conscientiously, mining, refining, and trading of critical raw materials would create employment, income, and an environmentally and socially equitable society. However, the history of mining has been embellished with complicated relationships with fragility and corruption, consequently spearheading conflicts. These conflicts are attributable to unfavourable policies, unaccountability, fragility, corruption, and retrogressive diplomacy by the government, nonchalance, dubiousness, lack of compliance, inordinate political influence and the exploitative tendencies shown by mining companies and investors, the dissatisfaction and outcry by activists demanding justice and equity, and worse still, the outrage expressed by citizens with abused human rights, usually in varying degrees of violence.

Indeed, no geopolitical zone worldwide is totally absolved of internal conflicts; however, zones possessing lots of CRMs are awash with crises (Fig. 4b). In view of this, we would like to present anecdotes of CRM-fuelled conflicts in selected geopolitical regions worldwide:

*The Americas* — The Americas is blessed with CRMs spanning arguably every latitude (Fig. 4b); hence, it is not unreasonable to assume they might be likewise impacted by waves of conflict. Canada has been long regarded as the behemoths of mining industry, with not less than three quarters of the world’s mining companies currently worth hundreds of billion dollars headquartered therein. However, this gargantuan status had been achieved by the displacement of far more than 600 Aboriginal communities in the world’s largest conserved Boreal Forest, which caused frictions between the natives, mining companies and the government. Although solutions have been proffered (albeit slowly adopted) to tensions arising from human rights and environmental violation, nationally, in the last two decades (IBCC-CBI 2008), Canada has not been totally exonerated of their incitement of fragility and corruption on the international scene. Contrary to a layman’s inference from the scenario Fig. 4b portrays, Canadian mining corporations have been responsible for shunting the consciousness of sovereign nations where their assets are domiciled exclusively towards the financial gains of mining operations, thereby neglecting the essentials. Worse still, the partnership and participation of local authorities legitimizes and endorses the ruthlessness of the Canadian asset holders in exploiting labours, environments and local communities, as witnessed in Latin America. For instance, a 16-year review of Canadian influence in Latin America mining (2000–2015) by the Justice and Corporate Accountability Project (JCAP) revealed that 28 Canadian companies were implicated in conflicts that resorted to 44 deaths (30 premeditated), 403 injuries (363 occurring during heated disputes), and 709 cases of criminalization, especially legal complaints, arrests detention, and charges (Imai et al. 2017). According to the Global Atlas of Environmental justice (Accessed 26 July 2022), the USA has endured local conflicts (especially bordering on social and environmental injustice) between some CRMs-mining companies and the community in episodes, such as Big Sandy lithium project in Arizona, Rio Tinto’s Kennecott Copper Mine in Utah, Twin Metals Minnesota Mining in the Superior National Forest, Thacker Pass lithium mine in Nevada, and the Hudbay Minerals copper mine in Santa Rita Mountains, Arizona, which has led to the suspension of some projects (www.ejatlas.org). In this regard, the USA, being fully cognizant of the greater social and environmental risks involved in mining CRMs, has been coy on full-scale CRM extractions across the country and instead outsourced its supply to other international CRM strongholds, particularly China, which supply well above 50% of their start-up materials.

However, as a consequence of what appears to be unfair trade policies, trade disputes, child and forced labor, and the financing of infrastructure that fuels civil unrest and political division in highly vulnerable and corrupt regions, the USA has decided to take more decisive actions to achieve self-sufficiency in CRMs. This initiative was initially launched during the Trump-Pence administration (Humphries 2019) and is now being continued under the Biden-Harris leadership (Newburger 2022). Consequently, we anticipate a significant increase in conflicts, particularly if the USA pursues the acquisition of CRMs in an irrational and illicit manner. In relation to conflicts, Latin America has experienced the most severe disturbances, both within the Americas and globally (Andrews et al. 2017). The conflicts in the region are partly linked to the extraction of CRMs for green energy and storage. Notably, four out of the top five countries worldwide prone to conflicts are located in Latin America: Chile, Guatemala, Mexico, and Peru. Chile has faced conflicts stemming from the mining of copper, lithium, and silver, some of which have persisted since the early half of the last century (Reisch 2023). These conflicts, such as the Las Vizcachitas Mining Project, Proyecto Minera Dominga, Zona de Sacrificio, Pascua Lama mine interruption, and Proyecto Expansión Andina 244 CODELCO, *inter alia*, have been triggered by the environmental, health, and socio-economic impacts of the projects. They often involve the intervention of state defense councils and litigation, leading to project interruptions or decommissioning (www.ejatlas.org). In Nicaragua, conflicts have arisen in locations such as Lote Ocho–El Estor, Proyecto Minero El Escobal, La Puya resistance, and Proyecto Minero Los Chocoyos. These conflicts involve forced evacuations, sexual violence, and murder, particularly in the vicinity of the Lote Ocho nickel mines. Local communities believe that such social atrocities occur with the approval of mining companies and their subsidiaries. As a result, they have engaged in protests and initiated lawsuits against these activities (Sveinsdóttir et al. 2021). Mexico has witnessed several conflicts related to the mining of manganese, silver, iron ore, lead, and zinc. Many protests have ranged from moderate to high intensity, often involving violence and arrests. These conflicts arise from aggressive and illegal land acquisitions through threats of violence, as well as environmentally irresponsible mining practices (as observed in cases such as Mina Dolores and La Colorada mining project) (Muñoz et al. 2022). In Peru, local communities have expressed dissatisfaction with substandard environmental, health, and socio-economic conditions through widespread protests, leading to confrontations, injuries, deaths, and arrests. For example, the infamous clash in Las Bambas between protesters and state police originated from a protest march, resulting in three deaths and fifteen injuries. Other grievances have been expressed through blockades and strikes, affecting mines such as the MMG-operated Las Bambas mine, Glencore-operated Antapaccay copper mine, as well as the Glencore- and BHP-operated Antamina copper and zinc mine in the past two years (Purdy and Castillo 2022).

*European Union (EU)* — As it stands, mining initiatives among member states of the EU continues to be a cog in the wheel of political and environmental agendas, especially in the emerging regional economies. A resuscitation and accentuation of mining projects has been observed, which have brought along with them intense pressures on ecosystems and human communities within the regions of mineral extractions (Kotilainen 2018; Schilling et al. 2018). Regrettably, these pressures lead to conflicts that endure every phase of the life cycle of a typical mining project, due to diverse impacts felt thereof (Kivinen et al. 2020). Just as was observed in other geopolitical zones, Kivinen et al. (2020) reported that recent mining conflicts in the EU were traced to socio-economic, environmental, and health concerns, where many projects were resisted during planning phase. A high-percentage of medium intensity conflicts (54%); however, there were moderately few high-intensity cases (14%), sparsely distributed in location and time. Agitators at the forefront of conflicts observed included neighbouring residents, social movements, local scientists, professionals, and farmers, where measure meted by the aggrieved included lawsuits, judicial activism, public campaigns, official complaints letters, and petitions as well as media-based activism. Sweden, Finland Serbia, and Spain have partaken in medium to high intensity conflicts, some which have gone violent and led to arrests. As of 2020, the Kallak Iron Mine in Sweden had still vehemently withstood the Beowulf Mining Company, which had been granted rights by the Bergsstaten Swedish Agency in 2006, from proceeding, while the Swedish government was advised by the UN to rescind orders given to Rönnbäcken nickel mine to proceed the proposed mining of nickel, cobalt, and magnetite in Sámi-populated community and its environs (Wilson and Allard 2023). In Finland, conflicts surrounding the Talvivaara nickel mining company, which extracted nickel, cobalt, copper, and zinc, resorted to the massive fines levied on company executives due to severe environmental pollution (Kivinen et al. 2020; Eerola 2022). The Rio Tinto proposed lithium mine in Jadar Valley, although backed by the Serbian government, has been challenged by medium-intensity conflict enactments, most of which challenge the decree made by the government and also object the recommendations of the environment impact assessment (EIA) reports (Stefanović et al. 2023). Last but not least, 2016 was not a great year for high directors of First Quantum, a Canadian firm, who was prosecuted for their negligence and subsequent large-scale damage to aquifer in Andalusia, which was traced to the Las Cruces copper mine they operated (Ecologistas en Acción 2019).

*Africa* — Significant reserves of critical raw materials and REEs deposited in several African nations are being realised, especially due to growing of foreign interest. However, from the context of mineral-fuelled conflicts, the African continent has and is still witnessing dramatic episodes in its campaign for resource control, socio-economic, and socio-political equity. In consequence, not less than 60% of the continent is affected by intense corruption and fragility, where sub-Saharan Africa is particularly implicated (Fig. 4b, supra). Essentially, conflicts in this region are observed under broad categories, such as civil society and state actors versus mining companies, trade unions versus mining companies, and artisanal miners versus industrial mining companies. In the first instance, there is often a more confrontational strategy deployed by the community members (led by secular and religious influencers) who do not have well represented institutions and material resources for legal actions. Frustration and consequent confrontation by this group of people are stirred up when certain culturally, spiritually, or religiously revered places are adversely impacted by mining operations or are at the verge of being encroached altogether. However, in cases involving taxes, profits and rents, government parastatals, municipal councils, and local politicians are swift in taking the reins. These aforementioned scenarios emphasize the asymmetric dynamics of governance among the government-mining company-local community trifecta, which has been observed in Ghana, Democratic Republic of Congo (DRC), Sierra Leone, Zimbabwe, and South Africa (Engels 2016, and references therein; Maguwu 2017). Conflicts between trade unions and mining companies could be incited by the unfair furlough and other socio-economic consequences resulting from privatization of state-owned mining companies, which leads to protests spearheaded by trade unions, non-governmental organizations, and sometimes academic scholars and civil society organizations (Larmer 2005; Rubbers 2010). Conversely, relationships between liberation and social movements, workers’ groups, and trade unions become fragile and tense, especially when distrust and betrayal are perceived. A memorable example occurred after a strike not backed by a trade union degenerated to clash between aggressors and state police as well as Lonmin security guards, leading to the death of 34 miners and 78 injured at the Lonmin-operated platinum mine in Rustenberg, South Africa (Alexander 2013). Depending on the agenda of the mining companies and, to some extent, the level of protection received from the government and political kingmakers, relationship between artisanal miners and industrial mining companies could be either mutualistic or antagonistic. Artisanal miners are most times are the earliest active discoverers of resource deposits; they mine, usually without licenses, for subsistence and might co-exist with small-scale farming and animal husbandry. However, unhealthy competition may set in when industrial mining companies deliberately apply for and are granted licenses to explore, full-scale, where artisanal miners have already established an indication of resource deposits, which is followed by displacement without compensation, leading to conflict. Due to informality and illegality of artisanal mining, miners have no rights, compensation, or legitimate access to political institutions and legislature during the commission of a mine project and have to rely on formally organised demonstrations or spontaneous marches and sabotage (Engels 2016). In other cases, where industrial mining companies brazenly corrupt, they may employ the services of artisanal miners, where the latter is short-changed during the sale of resources, which they had bought cheap from them (usually in exchange for weapons). Furthermore, industrial mining companies, expatriates, and political helmsmen might be implicated as undercover proponents of political instability civil unrest. This is because they may approve of these circumstances as a strategy to illegally explore and claim more resource-rich landmass of communities abandoned due to insecurity concerns. Iconic examples would include the frequent occurrence of social volatility and illegal mining in some resource-rich areas of Nigeria, DRC, Angola, Sierra Leone, and Guinea, *inter alia* (Church and Crawford 2018; Ogbonnaya 2020).

Aside from local conflicts, recent ramp-ups in demand for CRMs as well as the control of resource flow might lead to strained international relations among some countries. As of now, China leads in the international CRM scramble as it vivaciously and aggressively expands its reserves through mining and import licensure for certain critical minerals in Africa, Latin America, and Afghanistan (Umbach 2022). However, due to the global mandate for a switch to RES, the availability of these CRMs would shape the future of geopolitical conflicts, especially in deciding the congregation of allies and adversaries: A snippet of this can be observed through the activated Russia-Ukraine crisis and the soon-to-be China-Taiwan crisis, which the USA and EU have been implicated and might have untold ripple effects on the rest of the world. This is because viable substitutes are yet to be discovered for these materials, which would afford comparably excellent performances when integrated in the design of RES. In summary, considering the aforementioned issues only further emphasizes the farfetchedness of RES as an entrée to clean sustainable energy; hence, it warrants fresh perspectives on RES that would engender the seamless fusion of our present world’s perceptions of sustainable development.

## MFC: impetus for a circular bio-enabled economy and clean, sustainable energy

**Fig. 5:**
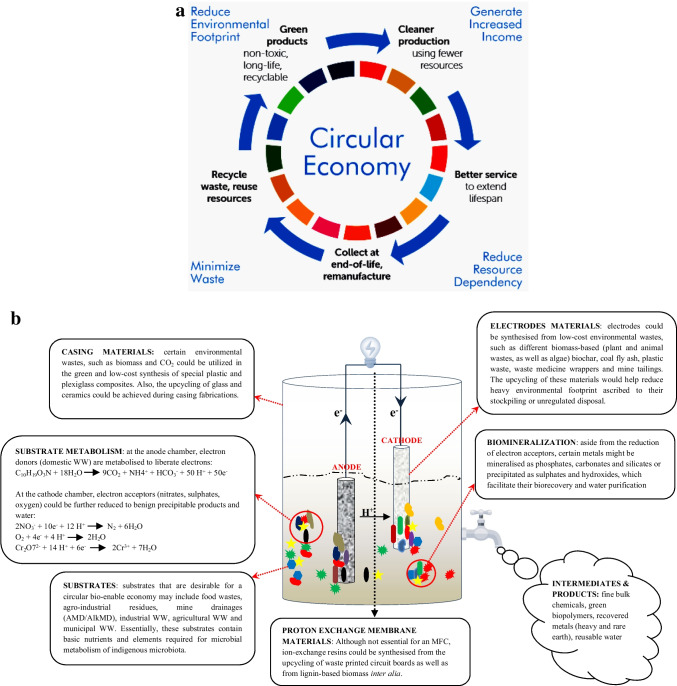
**a** The blueprint of a circular economy and its philosophies with respect to environmental sustainability (source: UNIDO 2017). **b** Outline of a microbial fuel cell (MFC) portraying its role in ensuring circular bio-based economy

The concept of a circular economy has indeed been visualized as the most critically beneficial thoroughfare to a sustainable society, on all fronts. This is because it is buoyed by four fundamental philosophies: waste minimization, environmental footprint deceleration, natural resource conservation, and economic innovation (Fig. 5a). From a bio-enabled economy perspective, we imply the facilitation of process circularity by bio-based agents, either as feedstock (carbon-based biomass) or as catalysts (microorganisms and their metabolites). Since the inception of this concept, major strides have been made to integrate bio-enabled economy into major sectors that provide the necessities for human development, such as energy, agriculture, and environmental sanitation. For instance, in agriculture, wastes have undergone biotransformation to produce fertilizers and organic matter, which enrich the soil and enhances biochemical and mechanical properties. Likewise, in energy, biomass has been valorised into energy-dense materials through diverse thermochemical pre-treatments techniques that often yielding high-value chemicals and volatile substances. In the environmental sanitation space, microorganisms have driven the biorecovery of various resources from waste streams, aside from the metabolites and fine chemical they might secrete thereupon. Interestingly, there are certain systems, whose assemblage and performance might involve adaption of feedstock, catalysts, or both, as major components for effective output; here, particular focus is devoted to MFC.

MFC is a new generation bio-enabled electrochemical system that facilitates the derivation of chemical metabolic energy from organic and inorganic substances and its subsequent conversion into electrical energy. For optimal performance of a typical MFC, the basic components are as follows: Electrodes, exchange membrane (optional), substrates, and microbial community must be intelligently selected or synthesized. Although works done earlier on synthesis, assemblage, and operation referred the use of simple organic substrates (glucose, acetate), electrodes from conventional composite components (platinum, palladium, silver, copper, graphite) (Colichman 1963; Austin 1967; and references therein) which are often expensive, contemporary investigations have extended focus to cheaper heterogenous sources of substrates as well as feedstock for electrode synthesis (Palanisamy et al. 2019; Moradian et al. 2021; Zhu et al. 2022a, b). As a consequence, MFC not only has immense potential to be the most feasible sustainable RES, but also thrives better on the upcycling of waste resources/streams (usually from other RES) than the other systems (Fig. 5b).

For instance, during the combustion of waste biomass and coal for heat and energy, largely underutilized residues are generated; adopting these wastes in the synthesis of functional electrochemical composites for MFC electrodes is an excellent approach to decongest process flow as well as limit on-site land encroachment or pollution. In a similar vein, aside from the utilization of certain waste streams as sources of microbial metabolic energy, certain valuable materials, chemicals, and resources could be recovered from wastewater passaged in an MFC. This has been evinced in a few examples among several reports on the recovery of nutrients (Shahid et al. 2022), critical raw materials (Işidar et al. 2019), and energy fuels (Munoz-Cupa et al. 2021). Interestingly, in scenarios where performance of MFCs begin to wane, the components can be inexpensively retrofitted for enhanced performance, recycled, downcycled, or repurposed altogether, which ensures its circularity. Typical examples of these phenomena can be observed for biochar electrodes, which can be subsequently used for soil amendment, gas storage, and segregation (Patwardhan et al. 2022); coal fly ash electrodes, which can ultimately be utilized as material components for construction purposes (He et al. 2021); as well as the utilization of spent substrates as biosolids from which fuel and other resources can be derived (Awasthi et al. 2022). Therefore, given that the competition for the unevenly distributed virgin mineral resources needed by other RES overruns that of the relatively untapped perpetually abundant and ubiquitous waste streams adopted for MFC, it is not imprudent to assume the latter is better positioned to enact the renewable and sustainable energy initiative. Likewise, since MFC is a strictly bioelectrochemical process, issues regarding atmospheric pollution are combated therewith, hence making it the closest phenomenon to clean energy. Altogether, we believe MFCs might increasingly grow in appeal and traction because it is consolidated by the following ideologies:

- *Ethics*: It is a general responsibility to adopt a green behavior, due to the bitter-sweet realities stemming from industrial advancement. MFCs are an insignia of the revolt against climate change and environmental degradation.
- *Environment*: MFCs might be pivotal in ensuring waste management, overturning the trend of water scarcity and restoration of biodiversity, as well as be an alternative energy source to the environment-fouling fossil fuels.
- *Socio-economy*: MFCs might serve as a two-edged sword in combatting economic losses resulting from active treatment of environmental waste and energy security. It also has the tendency to bridge extant gaps between gender, race, and social caste in the quest for financial stability through disruptive innovation.
- *Technology*: MFCs might ensure the irreversible momentum toward clean, sustainable energy. Conversely, it is bound to lead to other breakthroughs in biotechnology. Further research on MFCs might also afford the design of patent-worthy technologies.
- *Politics*: Sustainability is pan-partisan and is becoming a crux in political decisions and relations, worldwide; soon, it might be realized as the bedrock of sovereignty. MFCs have the potential to steer nations towards fulfilling UN SDG and other pledges that ensure sustainability, equity, and justice.
- *Society*: MFCs might be able to promote parity in access to the basic needs of life (e.g., clean safe water and electricity) among the different castes of society.

## Limitations of real-life MFC installations

MFC, if produced and installed using cheap environmental wastes, has the bright prospects of excelling on all fronts of sustainability, as it is not registered on the ledger of socio-political conflicts and environmental instability. However, it records heavy deficits in its transition to commercial-scale, real-life installations. MFC commercialization has indeed been hampered by some bottlenecks (Selvasembian et al. 2022), which have been identified at laboratory scale, and they border on the cell design, material configuration, size and spatial orientation of electrodes, metabolic diversity and electrogenicity of microbial community, energy production and conversion rate, and most importantly, the electrical environomics of the process. Scaling up involving commercialization for real-life applications implies the protraction of design as well as need for materials. The use of expensive materials, such as platinum and palladium and silver as electrodes, would not be environomically prudent, as they involve high financial and environmental costs during extraction, processing, and fabrication. For perspective, a commercially available electrode with dimensions of 0.5 mg/cm^2^ 20% platinum on carbon paper of 20 cm^2^, which is sparingly fitting for a laboratory scale MFC (250 mL) costs about US$ 250 (Fuel Cell Earth, USA). In spite of their high costs, some commercial electrodes (e.g., carbon-based materials) portray a smooth surface with minimal electrochemical activity and biocompatibility (Moradian et al. 2021). Moreover, the reaction (corrosion) of these materials with certain chemicals and salts in real-life aquatic habitats makes them impracticable for certain mesocosms, as they would contribute to loss of biodiversity therein; hence, the use of cheaper, chemically inert materials is encouraged. Challenges encountered in MFC performances include low power densities and high internal ohmic resistances, stemming from their structural configuration as well as electrolyte flux and dynamics. For example, MFC architectures with compartmentalized or far-apart electrodes have been shown to influence high internal ohmic resistance, reversal voltage, and reduced power densities (Fadzli et al. 2021; Dwivedi et al. 2022a). Substrate availability and their corresponding microbial assimilation are other major challenges faced by MFC modules, especially those modelled for large-scale, real-life applications pertaining to wastewater treatment. Substrate types and concentration play a pivotal role in energy generation, whereby suboptimal concentrations and overfeeding of substrates might obstruct the flow of biochemical energy in anodic compartment of an MFC module. This is because substrates influence the survival and interaction of metabolically active microbiota in anodic compartment, especially if real wastewater is used as substrate. In this regard, certain operational conditions and phenomena, such as pH, temperature, limiting nutrients, and co-metabolism, might result in voltage fluctuation or reversal. For instance, at conditions that stimulate methanogenic activity, anodic substrates and their electrochemical derivatives are scavenged; particularly, hydrogen (proton) that is crucial for balancing the electric charge at the cathode is consumed, thereby resulting in voltage reversal (Rajesh et al. 2015; Kim et al. 2020). In some cases, high salinity wastewater might prompt severe inorganic fouling, due to the blockage of precipitates, predominantly calcium (89%) and magnesium (7%) (Hiegemann et al. 2019; Guo et al. 2021). Also, fluctuations in wastewater intrinsic properties (especially homogeneity) which are caused by external influences, such as climate variation and flow pattern, and substrate heterogeneity could retard longevity of performance in the long run (Jadhav et al. 2021a, b). For instance, during nutrient limiting or adverse climatic and environmental conditions, competition for substrate intensifies, where electrogenic microorganisms may primarily either utilize the limiting substrate for essential cellular metabolism or synthesize biomolecules that would ensure their sustenance, thereby making electrogenesis secondary and thus reducing the electrical output of the cell. In this regard, the limitation posed by the microbiota of the MFC can be further observed through undesirable biomass proliferation, especially the growth of non-exoelectrogenic microbial communities and their respective immobilization of nutrients through biofilms formed, which might also create inaccessibility of electrons to the anode (Thulasinathan et al. 2022). Therefore, it is imperative to understand models depicting the microbiological, biogeochemical, and electrochemical profiles and prospects of a given MFC module before embarking on large-scale, real-life resource valorization and recovery ventures.

## Progress: positive strides in MFC development

**Fig. 6:**
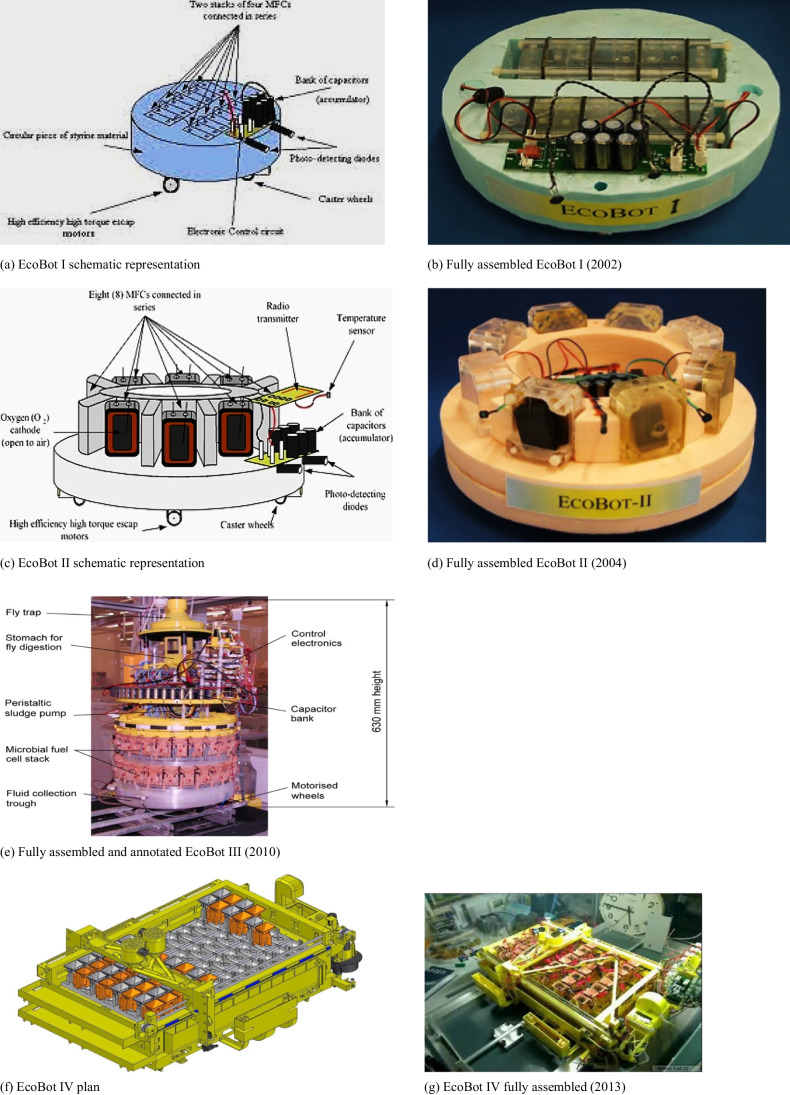
Models of EcoBot projects since the twenty-first century showing the gradual advancement in technology as well as waste treatment and energy generating capacity. Images were taken at the Bristol Robotics Laboratory, UWE courtesy Prof. Yannis Ieropoulos, from whom user permission was obtained

Early stages of MFC development were associated with a lot of challenges, mostly due to economic, structural, and engineering constraints that stymied the optimal performance of MFCs in simultaneous waste resources treatment and generation of electrical energy. As such, some researchers in MFCs development had advised that constraints particular to performance could only afford efficient waste resource treatment and recovery, at the expense of bioelectricity generation in practically unusable quotients (Logan and Regan 2006; Wang et al. 2015; Koffi and Okabe 2020; Yu et al. 2021). However, recent advances in the development and optimization of diverse aspects of MFC technology have warranted its reconsideration as a next-generation clean technology and also secure its long-term traction among researchers, industry, policy makers, and investors. In particular, footprints have been observed from positive strides in materials design, electromicrobiology, and power technology. In material design, conscientious efforts have been towards improving MFC structural configuration and coupling, as well as the synthesis of next generation electrochemical composites, through the beneficiation of certain agro-industrial and mineral residues (Zhang et al. 2019; Song et al. 2022; Zhu et al. 2022a, b). Our basic understanding of MFC configuration could be credited to primary setup described by Michael Potter (1911), which comprised a glass jar and a porous cylinder (also serving as an exchange membrane) in a concentric setting, individually plugged with platinum electrodes. Essentially, most MFCs are basically assembled as single chamber (SCMFC) or dual-chamber (DCMFC) modules, depending on preference, economy of scale, and end use. DCMFCs are the most commonly investigated, due to their unambiguous design and ease of sampling; however, their application has been restricted to laboratory scale, as well in microbial characterization and resource recovery, due to high internal resistance as well as the high cost associated with the exchange membrane required (Jadhav et al. 2021a, b). Moreover, its requirement of active exogenous supply of electron acceptors (notably oxygen) at the cathode makes it an energy intensive venture (Prathiba et al. 2022). Notwithstanding, appreciable progress has been made to augment the performance and reduce cost associated with DCMFC assemblage; typical examples can be observed in the algal biocathode, which substitutes the need for exogenous supply of oxygen, due to its respiratory oxygen supply during photosynthesis (Jadhav et al. 2017) and the adoption of ceramic materials as cost-effective and sustainable exchange membranes for use in large volume pilot-scale MFCs (Jadhav et al. 2022). Other recent developments that have been observed to improve the energy generating capacity of DCMFCs can be inferred from the reports of Gul et al. (2021), Hoang et al. (2022), Selvasembian et al. (2022), and references therein. SCMFCs are usually devoid of a well-defined cathode compartment; they instead mostly have their cathodes exposed for direct contact with atmospheric oxygen as an electron acceptor, thereby limiting the need for active aeration. They deliver an inexpensive approach in design and also in generation of electricity from wastewater treatment (Zhang et al. 2020); however, single SCMFC modules may prove cumbersome to operate, especially during resources recovery and routine cell maintenance. In spite of this, researchers have consistently improved on the configuration of MFCs with regard to spatial orientation of the electrodes and stacking of individual modules to amplify power generation capacity or even their scale down to miniaturized devices (Hoang et al. 2022; Prathiba et al. 2022). Moreover, MFCs have been further optimized and retrofitted in recent years to demonstrate their technological manipulability and adaptability, which has berthed the multi-technological variants, such as forward osmosis-MFC (FO-MFC) (Bhagat et al. 2022), membrane-MFC, constructed wetland-MFC (CW-MFC), sediment-MFC (Sed-MFC), phycological-MFC (Phyco-MFC), photo-MFC, and electrosorption-MFC (Song et al. 2022 and reference therein). Aside from the spatial orientation and configurations of cells, major strides have been likewise made in the synthesis of electrochemical composites as conduits for the generation of electrical energy. With peculiar regard to electrodes, recent research has been channelled to materials and techniques that would evidently measure up to the cardinal, desirable characteristics, which include surface area and sorption capacity, stability and durability, electrical conductivity, and cost and accessibility. This has been evinced by the evolution of electrode components from materials that are cost-intensive with intense environmental footprints, such as platinum, palladium, silver, copper, and graphite, to environmentally and economically friendly residues, such as biochar from agro-industrial leavings, sewage sludge, recalcitrant plastics, and other low-value, high environmental impact waste materials. In a similar vein, residues from minerals processing, such as coal fly ash, are being valorized for diverse environmental and energy-related applications. Moreover, the advent of the nanomaterial synthesis from these waste aggregates has made them liable to achieve cardinal characteristics of electrode better than any other group of materials. For example, a review of investigations by Kaur et al. (2020), Kamali et al (2022), and Wilberforce et al. (2022) has further elucidated the synthesis step, biocompatibility, and electrochemical capacities of low-cost and environmentally friendly nanocomposites, which we believe offers a promising outlook for future applications. Interestingly, recent advancements regarding precision in electrochemical cell design and its components have been made possible by additive manufacturing technologies (3D printing), which are anticipated to augur novel, custom-made MFC scaffolds and reactor designs (Dwivedi et al. 2022b). Furthermore, it facilitates their fabrication at points of demand, use, and convenience, which might in the long run effect cutbacks on energy, economic and environmental footprints associated with transportation of industry-fabricated MFC components and reactors. While MFC research was still in infancy, knowledge of microbially directed electrogenesis was limited to the use of mediators — electron shuttling molecules that supply electron acceptors or electrode surface with metabolically released bacterial electrons, as demonstrated by Michael Potter (1911) and other succeeding researchers up till the mid-twentieth century. Further advancement led to the discovery of microbial electron transfer without mediators by *Shewanella putrefaciens* IR-1 (Kim et al. 1999), which spearheaded a series of studies on pure cultures of model electroactive species (*Geobacter* sp. and *Shewanella* sp.) in the early twenty-first century. Major research has been on understanding the mechanisms, microbial machinery, and molecules involved in extracellular electron transfer (EET) as its implications in biotechnological applications (Nealson 2017). Consequently, the once indistinct aspects of this phenomenon have been unveiled and have been protracted to encompass a broader spectrum of microorganisms, notably the cable bacteria, as well as archaea, which has been observed to be a strategy for evolutionary adaption in diverse and extreme environmental niches (Lovley and Holmes 2022). Moreover, the interaction of phylogenetically diverse electroactive microbial communities of the aforementioned niches has been elucidated, especially as it applies to microbial assembly and function in electrode biofilms in an MFC (Lovely and Holmes 2022; Paquete et al. 2022). This new knowledge has proved helpful in the recent molecular manipulations of individual species, microbial communities, and biofilms for greater MFC performance (Choudhury et al. 2017; Das et al. 2022; Dwivedi et al. 2022b; Turco et al. 2022; Wang et al. 2022). Power technology is also a crucial merit that influences the commercial traction of MFCs, as it could determine their energy output and security. MFCs have constantly been challenged with reduced power output due to ohmic overpotentials and voltage reversals. Recent advancements in the development of power management systems have enabled a circumvention of the drawbacks faced. For instance, recent literatures by Prasad and Tripathi (2022), Dutta et al (2022), and Mukherjee et al (2022) give a historian account of the components, modalities of operation, and real-world applications of emerging PMSs, with case studies that emphasize the capabilities of such systems to upend energy insecurities and intermittences. Ultimately, in a world directed by the fourth industrial revolution (4IR), MFC seems to be relentlessly compliant or amenable to its components, especially artificial intelligence (AI). AI has been used in formulation of mathematical models, which are made to fit existing data and thereafter accurately predict future outcomes after cross validation; in this regard, models such as artificial neural network (ANN), deep neural network (DNN), and relevance vector machine (RVM), *inter alia*, have been deployed to optimize various aspects of MFC assemblage and performance. Moreover, AI algorithms had also been used to design automated, robot-facilitated MFC systems (EcoBot I, II, III, and IV; Fig. 6), which imitates functional ecosystems, albeit in artificial mesocosms, for enhanced energy production as well as environmental monitoring in real-time (Dwivedi et al. 2022b). Therefore, we surmise that MFC might gain increased relevance per advancement in techno-economic ecosystem, as long as the desire for a green sustainable environment is maintained.

## Reflections

Based on the aforementioned observations, it is evident that the transition towards renewable and sustainable energy cannot completely exclude fossil fuels indefinitely, nor can it be rushed. This is because fossil fuel technologies currently possess the most established and efficient means of achieving economies of scale and widespread applications. Additionally, recent geopolitical crises involving fossil fuel giants have reminded unprepared regions of the potential hardships that energy crises could bring if the majority or all of their energy need to rely solely on emerging renewable energy technologies. Undoubtedly, fossil fuels have had detrimental effects on climate change. However, completely disengaging from the fossil fuel sector cannot be hastily implemented, as their byproducts also serve as essential raw materials for critical petrochemicals. It is instead suggested that the proportion of fossil fuels in the energy sector be gradually reduced as emerging technologies reach maturity. Furthermore, existing fossil fuel processing plants should be retrofitted in alignment with the goals of a circular economy. This would promote the downcycling or upcycling of waste byproducts, aiming to minimize waste and maximize resource efficiency.

We also discussed the significant progress made by various emerging renewable energy systems in reshaping the dynamics of energy distribution and the overall energy sector. However, based on the issues we highlighted, including the increasing demand for finite critical raw materials (CRMs) and the accompanying environmental and social injustices, we conclude that these renewable energy systems are currently unable to fully embody the principles of true sustainability, nor are they strategically positioned to align with the goals of a circular economy. Therefore, it is crucial to focus research efforts on improving the production of components used in these systems. Specifically, there should be a pursuit of environmentally friendly and reusable materials. Additionally, it is essential to integrate individual renewable energy systems into modularized energy ecosystems to address the persistent problem of intermittence, which is a major drawback of weather-dependent energy systems. By doing so, we can mitigate the frequent disruptions caused by unpredictable weather conditions. In summary, to overcome the limitations and challenges associated with emerging renewable energy systems, we recommend enhancing the production of environmentally friendly and reusable components and integrating various systems to create a modularized energy ecosystem that can address the issue of intermittence.

Microbial fuel cells (MFCs) possess enormous potential to facilitate a seamless transition from fossil fuel–based energy to clean and sustainable energy sources. Additionally, they can help mitigate the environmental impacts associated with certain other renewable energy systems. This is primarily due to the widespread availability and abundance of two key components: inexpensive waste materials and biological organisms such as microorganisms and plants. Consequently, the implementation and utilization of MFCs involve the valorization of waste resources through upcycling or downcycling processes, the exploration of novel and valuable biotechnological capabilities of microbial communities, the recovery of critical raw materials, and the synthesis of environmentally friendly catalytic chemicals. Furthermore, the integration of MFCs within the energy sector is expected to promote equity and harmony in social, environmental, political, and economic aspects, aligning with the long-term goals outlined in the United Nations Sustainable Development Goals (SDGs). By incorporating MFCs, we can strive towards a sustainable future characterized by a green environment and clean energy. Therefore, further research on MFCs is necessary to consolidate our existing knowledge and continue making advancements in energy output. Although the development of MFCs is still in its early stages, we firmly believe that it is progressing towards an irreversible momentum in the realm of clean and sustainable energy, ultimately contributing to the establishment of a circular bio-based economy.

## Data Availability

Sources of data collected have been mentioned in the text.

## Abbreviations

Alk/AMD: Alkaline/acid mine drainage
CE: Clean energy
CH_4_: Methane
CO_2_: Carbon dioxide
CO: Carbon monoxide
COP27: 27Th Conference of Parties of the UNFCCC
CRMs: Critical/controversial raw materials
CSP: Concentrating solar thermal plants
DCMFC: Dual-chamber MFC
DOE: US Department of Energy
DRC: Democratic Republic of Congo
EIA: Environmental Impact Assessment
EROI: Energy return on investment
GE: Green energy
GHG: Greenhouse gas
GWh: Gigawatt-hour
HCl: Hydrogen chloride
HF: Hydrogen fluoride
H_2_S: Hydrogen sulfide
IEA: International Energy Agency
IPCC: Intergovernmental Panel on Climate Change
IR: Industrial revolution
IRES: Intermittent renewable energy systems
MW: Megawatt
MWh: Megawatt-hour
MFC: Microbial fuel cell
NH_3_: Ammonia
N_2_O: Nitrous oxide
NOx: Nitrogen oxides
NWCC: National Wind Coordinating Committee
NZE: Net zero emissions
PV: Photovoltaic panel
RE: Renewable energy
RES: Renewable energy system
SCMFC: Single-chamber MFC
SDG: Sustainable Development Goals
SE: Sustainable energy
SME: Small- and medium-sized enterprises
SO_2_: Sulfur dioxide
TBtu: Trillion British thermal units
TWh: Terawatt-hour
UN: United Nations
UNFCCC: United Nations Framework Convention on Climate Change
WW: Wastewater
